# Characterization of the Nonpolar and Polar Extractable Components of Glanded Cottonseed for Its Valorization

**DOI:** 10.3390/molecules28104181

**Published:** 2023-05-19

**Authors:** Zhongqi He, Sunghyun Nam, Shasha Liu, Qi Zhao

**Affiliations:** 1 USDA-ARS, Southern Regional Research Center, 1100 Allen Toussaint Blvd., New Orleans, LA 70124, USA; sunghyun.nam@usda.gov; 2 School of Energy and Environmental Engineering, University of Science and Technology Beijing, Beijing 100083, China; liushasha@ustb.edu.cn; 3 Coordinated Instrument Facility, Tulane University, New Orleans, LA 70118, USA; qzhao@tulane.edu

**Keywords:** bioactive, cottonseed, nuclear magnetic resonance spectroscopy, ultrahigh resolution mass spectrometry, van Krevelen diagrams, valorization

## Abstract

Cottonseed is the second major product of cotton (*Gossypium* spp.) crops after fiber. Thus, the characterization and valorization of cottonseed are important parts of cotton utilization research. In this work, the nonpolar and polar fractions of glanded (Gd) cottonseed were sequentially extracted by 100% hexane and 80% ethanol aqueous solutions and subjected to ^13^C and ^1^H nuclear magnetic resonance (NMR) spectroscopy and Fourier-transform ion cyclotron resonance mass spectrometry (FT-ICR MS), respectively. The nonpolar (crude oil) extracts showed the characteristic NMR peak features of edible plant oils with the absence of ω-3 linolenic acid. Quantitative analysis revealed the percentage of polyunsaturated, monounsaturated, and saturated fatty acids as 48.7%, 16.9%, and 34.4%, respectively. Both general unsaturated fatty acid features and some specific olefinic compounds (e.g., oleic, linolenic, and gondonic acids) were found in the nonpolar fraction. In the polar extracts, FT-ICR MS detected 1673 formulas, with approximately 1/3 being potential phenolic compounds. Both the total and phenolic formulas fell mainly in the categories of lipid, peptide-like, carbohydrate, and lignin. A literature search and comparison further identifies some of these formulas as potential bioactive compounds. For example, one compound [2,5-dihydroxy-N′-(2,3,4-trihydroxybenzylidene) benzohydrazide] identified in the polar extracts is likely responsible for the anticancer function observed when used on human breast cancer cell lines. The chemical profile of the polar extracts provides a formulary for the exploration of bioactive component candidates derived from cottonseed for nutritive, health, and medical applications.

## 1. Introduction

Cotton (*Gossypium* spp.) is a global crop grown primarily for fibers used in the textile industry. Currently, the majority of the cotton crop value (85–90%) derives from cotton fiber [1,2]. Thus, the characterization and valorization of other non-fiber biomass byproducts are important parts of research on cotton production and utilization [3,4,5,6,7,8,9,10]. With the advent of glandless (Gl) cottonseed, an especially attractive target, the enhanced utilization of cottonseed has been a trending topic in recent years, as cottonseed is the major byproduct from cotton fiber ginning [2,5,6,11,12,13]. Whereas cottonseed is a natural resource of agrochemicals (e.g., fiber, proteins, carbohydrates, and lipids), its nutritional value is hindered by the presence of a toxic chemical (i.e., gossypol) in the conventional glanded (Gd) cottonseed [5,14]. The current strategies of valorization of cottonseed include (1) the development of novel Gl cottonseed varieties for food applications [15,16,17,18,19,20,21,22], (2) synthesis and formulation of biobased materials from traditional Gd cottonseed and its byproducts for nonfood applications [23,24,25,26,27,28,29,30], and (3) exploration of the bioactive components in cottonseed for nutritive, health, and medical applications [31,32,33,34,35,36,37]. While these bioactive components are assumed to be peptide fragments, polyphenolics, and/or flavonoids, the specific functional groups or compounds are yet to be identified [33,38,39,40,41].

^13^C and ^1^H nuclear magnetic resonance (NMR) spectroscopy and Fourier-transform ion cyclotron resonance mass spectrometry (FT-ICR MS) are powerful tools in agrochemical and natural products research [42,43,44,45,46,47,48,49]. ^13^C and ^1^H NMR spectroscopy have been used to evaluate the functional groups of valorized cottonseed products, such as edible cottonseed oil [50], cottonseed oil-converted biodiesel [51], and cottonseed meal-based biochar [52]. FT-ICR MS has been applied to profile the chemical composition of cottonseed meal-derived bio-oil [53], waste cottonseed oil-based biokerosene [54], and the polar fraction of Gl cottonseed impacted by roasting [55]. Therefore, in this work, we applied these advanced instrumental techniques to characterize the nonpolar and polar fractions of Gd cottonseed. The primary objective of this work was to increase the basic knowledge of the chemical composition of the extractable fractions of Gd cottonseed. Through the chemical profiling, our purposes were to (1) confirm and/or identify the major nutrient and functional carbon (C) components in Gd cottonseed, (2) establish the potential bioactive capability of selectively identified compounds by literature comparison, and (3) highlight bioactive component candidates for future valorization research.

## 2. Results and Discussion

### 2.1. ^1^H NMR Spectral Features of Nonpolar Oil Fraction

**Figure 1:**
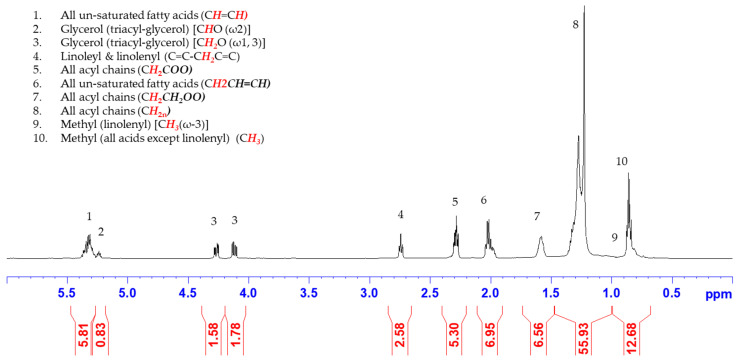
^1^H NMR spectrum of the nonpolar (oil) extract of Gd cottonseed. The integral peak ranges and values are presented at the bottom in red font. Functional peak proton assignments are based on [45,50,58].

The ^1^H NMR spectrum and relevant functional group proton assignments of the Gd sample are shown in Figure 1. These ^1^H NMR peaks appeared in 10 clusters ranging from 0.5 ppm to 5.5 ppm. The characteristic functional groups identified in the nonpolar extracts were the olefin peaks at 5.35 ppm, glycerol peaks at 5.25 ppm (methine) and 4.3 and 4.1 ppm (methylene), bis-allylic at 2.75 ppm, beta methylene at 2.3 ppm, allyl at 2.0 ppm, alpha methylene at 1.6 ppm, and long chain methylene at ca. 1.3 ppm. While the terminal methyl was apparent at 0.85 ppm, it is worth noting that another methyl peak at 0.95 ppm exclusively for the terminal methylene of ω-3 linolenic acid was not observed. Indeed, in the evaluation of the lipid profile of cottonseeds at different stages of development, Kurkuri et al. [56] reported that ^1^H NMR analysis demonstrated the presence of sterols (0.64 ppm), linolenic acid (0.95 ppm), and butyl ester of fatty acids (4.03 ppm) in the early stages. However, the ^1^H NMR peaks of the three compounds were not detected in the hexane extracts of late-stage cottonseed from nearly mature fruits with fibers opened up. The similarity of the ^1^H NMR spectral feature(s) to those of the hexane extracts of matured field cottonseeds [56] and other refine cottonseed oil samples [50,51,57] implied that the chemical composition of the 100% hexane-extracted nonpolar fractions of the Gd cottonseed was typical for a cottonseed oil sample. The quantitative comparison of the relative intensities of these peaks with edible oils further confirmed this observation, as these values of the Gd-n sample are more similar to those of cottonseed than other samples in the literature (Table 1). Furthermore, the data in Table 1 indicates that the oil fraction of cottonseed belonged to the same category as that of corn and peanut oils without ω-3 linolenic acid (i.e., peak 9), while the ω-3 lipid was present in canola, soybean, and walnut oils.

More specifically, the allylic region of Peak 6 at 2.00 ppm was present in all unsaturated fatty acids, and the two protons of the acyl group of Peak 5 at 2.30 ppm contributed one methylene group to all types of fatty acids. In other words, the relative intensities of peaks 4, 5, and 6 in the ^1^H NMR spectrum (Figure 1) reflect the ratios (i.e., relative distribution) of polyunsaturated fatty acids (PUFAs), monounsaturated fatty acids (MUFAs), and saturated fatty acids (SFAs) [58,59]. Thus, the integration ratio of Peak 6 over 2x that of Peak 5 represents the relative percentage of unsaturated fatty acids in the sample, so the relative distribution of SFAs was equal to 100%—the percentage of unsaturated fatty acids. Additionally, the protons in bi-unsaturated fatty acids were shifted to Peak 4 at 2.75 ppm due to their attachment to the bi-allylic carbon. Therefore, the integration ratio of Peak 4 over Peak 5 represents the relative percentage of PUFAs. Subsequently, the percentage of MUFAs was the difference between total unsaturated fatty acids and PUFAs. Table 2 lists the relative distribution of the three types of fatty acids in Gd cottonseed samples in comparison to those of other seed oils (hexane extracts) in the literature. While all four oil samples were highly unsaturated (65.6–98.0%), the comparisons indicate the fatty acid profile of Gd cottonseed was similar to that of Spondia mombin seed oil. While both walnut and bluebell oil samples showed a higher percentage of unsaturated fatty acids than Gd cottonseed, walnut was dominated by PUFAs, and bluebell was dominated by MUFAs. Similarly, Barison et al. [60] applied these ^1^H NMR spectral characteristics to develop a simple method for the determination of linolenic, linoleic, oleic, and saturated fatty acids. They found the ratio changes of these fatty acid compositions in oil samples of rice, sunflower, corn, canola, olive, and soybean. However, it is impossible to quantitatively compare those data with those in Table 2 not only due to the difference in the specific fatty types but also as no tabulated quantitative values were presented in [60].

### 2.2. ^13^C NMR Spectral Features of Oil Extracts

**Figure 2:**
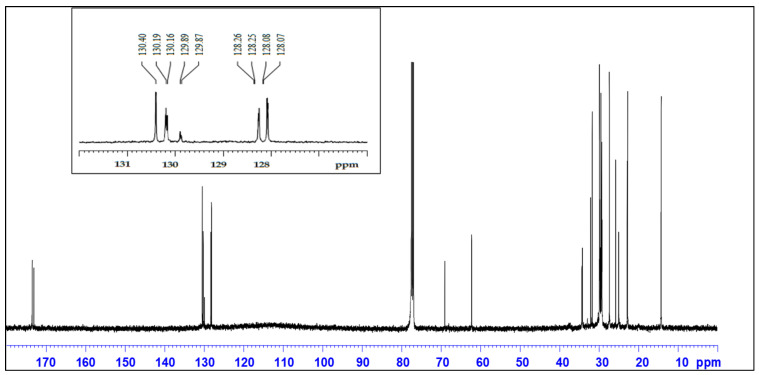
^13^C NMR spectrum of the nonpolar (oil) extract of Gd cottonseed. Inset is showing the peak details around 130 ppm.

Figure 2 shows the ^13^C NMR spectrum of the nonpolar oil fraction of Gd cottonseed. The ^13^C NMR spectrum was similar to that of cottonseed oil in the literature [51]. However, the previous work [51] did not give detailed peak assignments of this cottonseed oil sample. Thus, we assigned and discussed the ^13^C NMR of our sample based on the general ^13^C NMR spectral features of plant oils (e.g., olive, coconut, and soybean oil) [45,62]. By comparison, it was notable that the chemical shift values of the ^13^C NMR peaks in the oil fractions of our cottonseed sample were systematically 0.20–0.4 ppm higher than those in the literature [45,58,61,62]. Both the minor differences between the oil types [62,63] and the reference CDCl_3_ variability [64] could have contributed to the consistent minor upshift of those ^13^NMR signal peaks in our Gd cottonseed sample. With appropriate minor adjustment, those peaks in the Gd cottonseed sample could be assigned to the oil components of triacyl glycerol, oleyle, linoleyl, and other acyl-chain C-functional groups (Table 3). Those data further confirmed that the nonpolar hexane fraction of Gd cottonseed was a typical plant oil sample. An additional benefit of the ^13^C NMR analysis of the plant oil samples was the larger dispersion of some common unsaturated (olefinic) acyl groups around 130 ppm [63]. In other words, different unsaturated acyl carbons could be distinguished by the ^13^NMR spectral features in this region. For example, the *S. Mombin* seed oil shows 7 peaks from 127.88 ppm to 130.20 ppm, which are assigned to the linoleyl and oleyl functional groups [61]. Hama et al. [58] reported 11–15 peaks in four clusters from 127.03 ppm to 131.82 ppm for the hexane extracts (oil) of walnut. These peaks indicated the presence of several unsaturated fatty acids, such as oleic, linolenic, vaccenic, and gondonic acids. Similarly, the nonpolar extracts of Gd cottonseed showed 9 peaks in two clusters from 128.07 ppm to 130.40 ppm (Figure 2 inset). The distribution patterns of these peaks were comparable to the patterns of walnut oil but without the two end peaks at 127.03 ppm and 131.82 ppm (C15 and C16 linoleyl). These results implied that several polyunsaturated fatty acids isomers, but not ω-3 𝛼-linolenic acid, were also present in the cottonseed oil sample. More research needs to be performed to confirm the observation. In particular, spiking the samples with the referenced fatty compounds would be helpful for such a purpose [65].

### 2.3. ESI FT-ICR Mass Spectral Analysis of Gd Cottonseed Extracts

**Figure 3:**
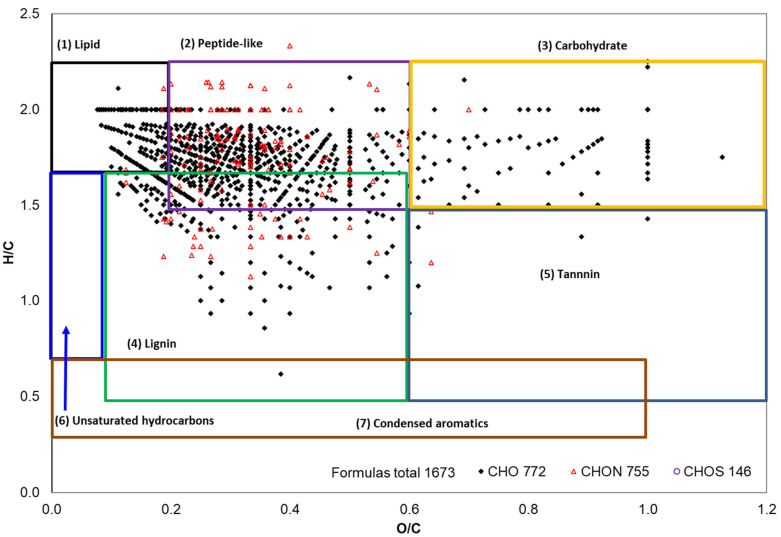
The Van Krevelen diagram of the formulas detected by ESI FT-ICT MS in 80% ethanol extracts of Gd cottonseed. Overlain boxes show where the seven major biomolecular compound classes align.

Both the nonpolar (100% hexane) and polar (80% ethanol) extracts of Gd cottonseed were subjected to negative ion ESI FT-ICR analysis. This ultrahigh-resolution mass spectrometry detected 1673 formulas in the polar extracts (Figure 3). Among them, 772 formulas contained CHO elements, 775 had CHON elements, and 146 had CHOS elements. Per the O/C and H/C values defined by V-K diagrams, these formulas were further separated into seven categories. Visually, those formulas were mainly in the categories of lipid, peptide-like, carbohydrate, and lignin. The relative diversity (number-weighted %) and abundance (magnitude-weighted %) of the formulas in the seven classes are listed in Table 4. The quantitative data confirmed the visual observation of the dominant four types of biomolecules in the 80% ethanol extracts of Gd cottonseed. These quantitative data also indicated that the diversity and relative abundance of these biomolecules were not in a consistent order among the seven categories. For example, the lignin category contained the most formulas (784) and accounted for 46.9% of the total diversity. However, its abundance was only 6.6% of the total identifiable mass of the extracts. The most abundant biomolecule was lipid (65.2% of mass abundance) but with only 165 different lipid formulas (9.9% of total formulas).

Furthermore, approximately 1/3 (1067 of 1673) of the formulas were potential phenolic compounds (Table 4). Almost all of the formulas classified as lignin or tannin were phenolic molecules, as multiple phenolic rings are the structural characteristics of these two categories of biomolecules. Thus, the two types of compounds accounted for 78.8% of the diversity and 72.4% of the potential phenolic compounds in the polar extracts of Gd cottonseed. There was a moderate presence of phenolic peptides, which accounted for 64% of the total peptide formulas. Lipid and carbohydrate were mainly present in the polar extracts as non-phenolic compounds, as only 42 of the 165 lipid formulas and 38 of the 172 carbohydrate formulas were potential phenolic molecules. Together, these accounted for 7.6% of phenolic compounds. However, their abundance was significant and represented 38.6% of the total phenolic mass abundance.

Table 4 compares the relevant data from Gd versus Gl cottonseed from previously published work [55]. The distribution patterns in the seven types of formulas for both the total and phenolic compounds were similar between Gd and Gl samples, with lignin as the dominant component. Compared to the Gl sample, however, both the total and phenolic formulas in the Gd sample were more than doubled. For example, the lignin formulas increased from 195 in the Gl sample to 784 in the Gd sample, a 4-fold increase. In addition to the extraction efficiency, some precursors and metabolites of gossypol may have contributed to the observation of the phenomenal difference of the Gd sample from the Gl sample while gossypol (C_30_H_30_O_8_, molecular mass 518.563 D) itself was not detected in the 80% ethanol extracts of Gd sample. The observed minor differences in peptide distribution were consistent with a previous observation of storage protein distribution in the two cottonseed samples (Gd versus Gl) [66].

### 2.4. Selected Potential Bioactive Compounds in the Polar Extracts of Gd Cottonseed

The ultrahigh resolving power of FT-ICR-MS not only allowed the assignment of its M/Z peaks to specific formulas but also further identified them to the relevant functional compounds and isomers of some formulas by literature comparison [46,67]. Through literature search and comparison, we identified the top 15 potential bioactive compounds with a double bond equivalent (DBE) ≥ 4 in the polar extracts of Gd cottonseed (Table 5). Thirteen of the 15 compounds were also identified in the Gl cottonseed sample, which was extracted in the same procedure, although their abundances were not equivalent. Three compounds (i.e., 2, 4, and 5) were observed to have >0.1% higher abundance in the Gd sample than in the Gl sample, but the other three compounds (i.e., 1, 3, and 11) showed >0.1% higher abundances in the Gl sample than in the Gd sample. The abundances of the other nine compounds had similar levels between the two types of cottonseed with differences <0.1%. These components (formulas) could serve as future test candidates to separate bioactive compounds in Gd and Gl cottonseed.

As a stable oxidation product of linoleic acid, hydroxyoctadecadienoic acid (HODE) was the top compound (in terms of abundance) in both the Gd and Gl samples, shown in Table 5. HODE isomers have demonstrated strong anti-inflammatory effects in a Murine macrophage cell model [68]. However, increased HODE levels could contribute to the progression of atherosclerosis and the risk of clinical events, such as a myocardial infarction or stroke [69]. 2,5-Dihydroxy-N′-(2,3,4-trihydroxybenzylidene)benzohydrazide was the third and second in abundance in the Gd and Gl samples, respectively, in the top 15 identifiable potential bioactive compounds (Table 5). It is a hexokinase 2 inhibitor [70]. Hexokinase 2 is required for tumor initiation and maintenance, and its ablation inhibits the neoplastic phenotype of human lung and breast cancer cells in vitro and in vivo [71]. During the evaluation of the effect of bioactive components from cottonseeds in human cancer cells, Cao, Sethumadhavan, and Bland [38] reported that the 80% ethanol extracts from cottonseeds decreased the mitochondrial activity of cancer cell lines derived from the breast and pancreas. While Cao, Sethumadhavan, and Bland [38] could not identify the inhibitory compound(s) via HPLC-UV-MS, it would be interesting to investigate if the 2,5-dihydroxy-N′-(2,3,4-trihydroxybenzylidene) benzohydrazide identified in this work contributed to the anticancer function of the cottonseed extracts. N-acyl amino acids were also prominently observed, and they have been highlighted for their therapeutic potential [72]. They are chemically related to the endocannabinoids and belong to the complex lipid signaling system known as the endocannabinoidome. While the three acylamide compounds were detected in the Gd samples, only one was detected in the Gl cottonseed with lower abundance. This information will aid the characterization and valorization of Gd and Gl cottonseed as functional food supplements and biomedical additives [31,38,73]. Manipulation of the sample processing could alter the abundance of some compounds. For example, roasting Gl cottonseed could increase the abundance of the hexokinase 2 inhibitor 2,5-dihydroxy-N′-(2,3,4-trihydroxybenzylidene) benzohydrazide but decrease the abundance of asperspin A (C_16_H_21_O_4_) [55].

**Table 5:** Identities and relative abundance (%) of 15 selected potentially bioactive compounds with a double bond equivalent (DBE) ≥ 4 in the polar extracts of glanded (Gd) cottonseed. The relative abundances of these compounds in glandless (Gl) cottonseed samples are also listed for comparison [55].

MS Peak (*m*/*z)*	Theoretic Mass	[M − H]^−^ Formula	DBE	Abundance (%)		Compound Name and Potential Function	Reference
				Gd	Gl [55]		
293.2123	293.2122	C_18_H_29_O_3_	4	0.609	0.787	Hydroxy-octadecatrienoic acid; anti-inflammatory	[74,75,76]
392.3173	392.3170	C_24_H_42_O_3_N	4	0.478	0.162	3-Methoxy-1-methoxymethyl-3-phenylpropyl)dodecanamide; ceramide trafficking inhibitor analogue	[77]
305.0779	305.0779	C_14_H_13_O_6_N_2_	9	0.382	0.625	2,5-Dihydroxy-N′-(2,3,4-trihydroxybenzylidene)benzohydrazide; hexokinase 2 inhibitor	[70]
426.3017	426.3014	C_27_H_40_O_3_N	8	0.291	ND ^a^	N-Docosahexaenoyl valine or N-linoleoyl phenylalanine; N-acylamides	[78]
378.3015	378.3014	C_23_H_40_O_3_N	4	0.233	0.070	N-linoleoyl valine; N-acylamides	[78]
290.0882	290.0881	C_11_H_16_O_8_N	4	0.204	0.138	Pyroglutamic acid hexose; bioactive metabolite	[79]
277.2173	277.2173	C_18_H_29_O_2_	4	0.155	0.182	Linolenic acid isomer; nutrient	[74,75]
309.2073	309.2071	C_18_H_29_O_4_	4	0.120	0.121	Hydroperoxy-octadecatrienoic acid, anti-inflammatory	[69,74]
278.0670	278.0670	C_13_H_12_O_6_N	8	0.117	0.208	N-coumaroyl aspartic acid; bioactive amino derivatives	[80]
499.3279	499.3276	C_27_H_47_O_8_	4	0.104	0.076	Cholestane octaol; Polar Steroid	[81]
387.1663	387.1661	C_18_H_27_O_9_	5	0.100	0.234	Tuberonic acid hexoside; tuber-forming substance	[82]
431.2288	431.2287	C_21_H_35_O_9_	4	0.091	0.048	Neorehmannioside; carotenoid glycoside	[83]
402.3015	402.3014	C_25_H_40_O_3_N	6	0.084	ND ^a^	N-palmitoyl phenylalanine; N-acylamides	[78]
319.0937	319.0936	C_15_H_15_O_6_N_2_	9	0.084	0.128	5-Phenyluridine; fluorescent nucleotide	[84]
307.1915	307.1915	C_18_H_27_O_4_	5	0.084	0.054	Dihydrocapsiate; thermogenic	[85]

^a^: Not detected.

## 3. Materials and Methods

### 3.1. Materials

The whole, mechanically dehulled Gd cottonseed sample was provided by Cotton, Inc. (Cary, NC, USA). The macro chemical composition of these Gd kernels has been investigated and is listed in Table 6 [14]. All other chemicals were reagent grade, purchased from Sigma-Aldrich (St. Louis, MO, USA) or Fisher Scientific (Pittsburg, PA, USA).

### 3.2. Sequential Extraction of Polar and Nonpolar Fractions of Gd Cottonseed

The sequential extraction of polar and nonpolar components from Gd cottonseed kernels followed the procedure reported previously [14]. Before the extraction, these kernels were ground for 3 min in a stainless-steel jar of a Waring Commercial Blender (Model WF2211214, Torrington, CT, USA). Ground Gd particles (5.00 g each) were then placed in a 50 mL centrifuge tube with 15 mL of 100% hexane and shaken at room temperature (26 °C) overnight (18 h) with a rotary shaker (60 rpm). Those tubes were then centrifuged for 30 min at 5 °C and 2500× *g* by a Centra GP8R (International Equipment Company, Needham, MA, USA). After centrifugation, the supernatant in the tubes was collected, and the residual pellets were extracted by 100% hexane one additional time via the same procedure. After the second extraction, the residual parts were washed twice with 100% hexane (5 mL each). The washed residual pellets were then twice subject to extraction by 80% ethanol (i.e., ethanol/water 4:1, 15 mL each) with the same procedure used for the 100% hexane extraction. The supernatants of the 100% hexane extracts were placed in a venting hood to evaporate the hexane out at room temperature (26 °C). The non-evaporated leftover was designated as the extractable nonpolar oil fraction of the cottonseed kernels. The supernatants of the 80% ethanol were dried in a vacuum (up to 25” Hg) oven at 40 °C to constant weights and designated as the extractable polar components (fractions) of Gd cottonseed.

### 3.3. NMR Spectral Analysis

The nonpolar oil extracts were analyzed by ^1^H and ^13^C nuclear magnetic resonance (NMR) spectroscopy at ambient probe temperature on a Bruker DRX 500 NMR instrument (Karlsruhe, Germany). Each sample was dissolved at 10% concentration (weight/volume) in deuterochloroform and placed in a 5 mm NMR tube. Standard instrument conditions were used, including a 30-degree pulse and a 5-s pulse delay. The chemical shifts were referenced to CDCl_3_ at 77.23 ppm for ^13^C NMR and the CHCl_3_ residues at 7.26 ppm for the ^1^H NMR spectrum [64]. The ^1^H NMR spectrum was normalized with the peak at 1.3 ppm as the maximum, and the ^13^C spectrum was normalized in reference to the aliphatic region (peaks around 30 ppm as the maximum) [45,56]. The quantitative calculation of the peak integral (i.e., relative peak intensity) was performed by Bruker Topspin 2.1 NMR software.

The relative distributions of polyunsaturated fatty acids (PUFAs), monounsaturated fatty acids (MUFAs), and saturated fatty acids (SFAs) in the nonpolar fraction of Gd cottonseed were quantified per their different functional group protons identified by the ^1^H NMR [58,59]. Specifically, with the peaks numbered as in Figure 1, the relative percentage (%) of the three types of fatty acids was calculated via the integrals of Peaks 4, 5, and 6 as follows: PUFAs = Peak4/Peak5; MUFAs = (Peak6/2 × Peak5) − PUFAs; and SFA = 1 − (Peak6/2 × Peak5).

### 3.4. ESI FT-ICR MS Spectrometry

Before the analysis, the dried sample of 80% ethanol extracts was diluted 100-fold using methanol. The ultrahigh-resolution mass spectral analysis was provided by the State Key Laboratory of Heavy Oil Processing, China University of Petroleum (Beijing, China), using the procedure reported previously [47,55]. Briefly, the sample was continuously infused into the Apollo II ESI ion source of a Bruker 9.4 Tesla Apex-Ultra FT-ICR MS spectrometer (Billerica, MA, USA), introduced by a syringe pump operating at 120 μL h^−1^. The lower and upper mass limit was set to *m*/*z* 150 and 2000, respectively. The spray shield voltage was set to 3.0 kV. The capillary voltage was set to 3.5 kV, and the capillary column end voltage was −320 V. Mass spectra were collected over 128 scans, with an ion accumulation rate of 0.02 s. MS peaks were detected in broadband mode at 200–800 *m*/*z*. The detection error was within 1 ppm absolute mass.

For molecular formula calculation, six elements, C, H, O, N, and S (C_5-50_H_5-100_O_1_-_30_N_0-4_S_0-2_), were taken into consideration using a self-written software routine [47]. The threshold value of the signal to noise (S/N) of the *m*/*z* molecular formula calculator was ≥6. Formulas were assigned based on a list of conservative rules that ensured that the formulas were chemically possible in nature [53]. In the vast majority of cases, the assigned formulas were within an error value of <0.5 ppm compared to the theoretic mass of the designated formulas. Double bond equivalents (DBE) values were calculated by the equation (2c + 2 + n + p − h)/2, corresponding to the molecular formula CcHhNnOoSsPp [46]. As at least one phenyl ring (DBE = 4) is present in a phenolic compound, those formulas with a DBE ≥ 4 were further considered as potential phenolic compounds in data discussion [55,67].

### 3.5. Van Krevelen (V-K) Diagrams of ESI FT-ICR MS Data

V-K diagrams are graphical plots of the elemental H/C versus O/C ratios of the molecular formulas, with the diagram space separated into seven discrete regions of biomolecular groups [47,86]. Both the total and potential phenolic formulas were subjected to the analysis. The seven V-K spaces (categories), with some overlap, were as follows: (1) lipid-like compounds in the O/C 0.0–0.2 and H/C 1.7–2.2 region; (2) peptide-like compounds in the O/C 0.2–0.6 and H/C 1.5–2.2 region, with N/C > 0.05; (3) carbohydrate-like compounds in the O/C 0.6–1.2 and H/C 1.5–2.2 regions; (4) lignin-like compounds in the O/C 0.1–0.6 and H/C 0.5-1.7 region; (5) tannin-like compounds in the O/C 0.6–1.2 and H/C 0.5–1.5 region; (6) unsaturated hydrocarbon in the O/C 0.0–0.1 and H/C 0.7–1.7 region; and (7) condensed aromatics in the O/C 0.0–1.0 and H/C 0.3–0.7 region. In addition, those formulas outside of the seven V-K spaces were labeled as “extra” or “other” [46,53].

## 4. Conclusions

This work sequentially extracted nonpolar and polar components from Gd cottonseed by 100% hexane and 80% ethanol aqueous solutions, respectively. ^13^C and ^1^H NMR spectra of the nonpolar extracts showed the characteristic peak features of edible plant oils with the absence of ω-3 linolenic acid. The relative distributions of polyunsaturated, monounsaturated, and saturated fatty acids were 48.7%, 16.9%, and 34.4%, respectively. In addition to the general classification (e.g., as olefinic) of the unsaturated fatty acids, the NMR data revealed the presence of specific types of unsaturated fatty acids (e.g., oleic, linolenic, and gondonic acids) in the nonpolar (crude oil) fraction. FT-ICR MS spectrometry detected 1673 formulas, with approximately 1/3 being potential phenolic compounds in the polar extracts. Those formulas were mainly categorized as lipid, peptide-like, carbohydrate, and lignin. The most abundant fifteen potentially bioactive compounds were identified through literature search and comparison, providing targets for the future exploration of the bioactive functions of the polar extracts. Notably, a specific chemical [2,5-dihydroxy-N′-(2,3,4-trihydroxybenzylidene) benzohydrazide] identified in the polar extracts may provide a clue to the identity of the bioactive compound(s) of cottonseed extracts that affect human cancer cell growth previously reported. The separation and recovery of such bioactive compounds from cottonseed promise to be a useful research topic in the valorization of cottonseed.

## Figures and Tables

**Table 1:** Comparison of the relative intensities (%) of ^1^H NMR functional group protons in nonpolar (oil) extracts of Gd cottonseed kernels (Gd-n) to those of edible oils reported in the literature. Refer to Figure 1 for the functional carbon group proton assignments.

	Chemical Shift Peak and Position in ppm											
	1	2	3	3	4	5	6	7	8	9	10	
	5.35	5.25	4.3	4.1	2.75	2.30	2.00	1.6	1.3	0.95	0.87	Reference
Gd-n	5.81	0.83	1.58	1.78	2.58	5.30	6.95	6.56	55.93	ND ^a^	12.68	This work
Cottonseed	7.41	1.03	1.99	2.09	3.21	6.09	8.71	6.46	53.99	ND	9.02	[50]
Corn	8.27	1.02	2.01	0.25	3.34	6.06	10.27	6.63	52.99	ND	9.15	[50]
Canola	2.36	0.34	0.64	0.65	67.71	1.93	3.57	2.03	17.79	0.28	2.70	[50]
Peanut	6.25	0.97	1.92	1.99	1.72	5.82	9.24	6.13	57.40	ND	8.56	[50]
Soybean	8.76	1.03	2.01	2.06	3.92	6.06	9.67	6.16	51.20	0.14 ^b^		[50]
Walnut	9.61	0.97	3.79 ^c^		4.76	5.73	10.39	5.83	48.06	1.17	9.71	[58]

^a^: Not detected. ^b^: Peak at 0.95 ppm detected, but data reported with peak at 0.87 ppm. ^c^: Data reported for both peaks at 4.3 and 4.1 ppm.

**Table 2:** Relative distribution (%) of polyunsaturated (PUFAs), monounsaturated (MUFA), and saturated (SFAs) fatty acids in nonpolar (oil) extracts of Gd cottonseed kernels (Gd-n) and those of seed oils (hexane extracts) reported in the literature.

	PUFAs	MUFAs	SFAs	Reference
Gd-n (Cottonseed Oil)	48.7	16.9	34.4	This work
Spondias mombin Seed	43.5	29.4	27.1	[61]
Walnut Oil	84.0	13.0	2.0	[58]
Bluebell Oil ^a^	11.0	79.6	9.2	[59]

^a^: Averages calculated per the five-year data in [59].

**Table 3:** Chemical shifts and carbon functional assignments of ^13^C NMR peaks in nonpolar (oil) extracts of Gd cottonseed kernels (Gd-n) according to the NMR studies of edible oils reported in the literature [45,58,61,62].

Chemical Shift (ppm)	Carbon	Assignment
14.25, 14.34	α-CH_3_	All acyl chains
22.75, 22.90	β-CH_3_	All acyl chains
25.00, 25.07	C-3	All acyl chains
25.81	C-11	Diallylic
27.39	C8-11 (oleyl), C8-14 (linoleyl)	Allylic
multiple 29.24–29.96 peaks	(CH_2_)n	All acyl chains
31,72, 32.11, 32.13	C-16	R-CH_2_-CH_2_-CH_3_ (stearyl, oleyl, linoleyl)
34.21, 34.24, 34,38	C-2	All acyl chains
62.29	α-CH_2_O	Glycerol (triacylglycerol)
69.06	β-CH_2_O	Glycerol (triacylglycerol)
128.07, 128.08	C-12	Linolenyl
128.25, 128.26	C-13	Linolenyl
128.89, 129.87	C-9	Linolenyl, oleyl
130.16, 130.18, 130.40	C-10, C-11, C-12	Linolenyl, gondoyl
173.05	α-C-1 Glycerol	Carbonyl (triacylglycerols)
173.46, 173.51	β-C-1 Glycerol	Carbonyl (triacylglycerols)

**Table 4:** Diversity (the number and % of formulas) and relative abundance (% magnitude) of the seven identified classes and one unidentified (other) class of total and potential phenolic formulas in the polar extracts of glanded (Gd) cottonseed samples. For comparison, data from glandless (Gl) cottonseed samples are also listed in the table [55].

Class		Gd		Gl [55]	
		Total	Phenolic	Total	Phenolic
Lipid	Number	165	42	111	30
	% Formulas	9.9%	4.0%	15.2%	9.0%
	% Magnitude	65.2%	14.5%	61.0%	10.7%
Peptide	Number	232	149	74	40
	% Formulas	13.9%	14.0%	10.1%	12.1%
	% Magnitude	1.7%	8.2%	1.7%	6.3%
Carbohydrate	Number	171	38	92	14
	% Formulas	10.2%	3.6%	12.6%	4.2%
	% Magnitude	6.7%	24.1%	7.6%	6.5%
Lignin	Number	784	765	195	186
	% Formulas	46.9%	72.0%	26.7%	56.0%
	% Magnitude	6.6%	61.9%	5.3%	57.7%
Tannin	Number	73	72	18	18
	% Formulas	4.4%	6.8%	2.5%	5.4%
	% Magnitude	1.1%	10.5%	0.7%	7.8%
Unsaturated Hydrocarbon	Number	1	1	0	0
	% Formulas	0.06%	0.09%	-	-
	% Magnitude	<0.01%	<0.01%	-	-
Condensed Aromatic	Number	0	0	0	0
	% Formulas	-	-	-	-
	% Magnitude	-	-	-	-
Other ^a^	Number	247	0	240	44
	% Formulas	14.8%	-	32.9%	13.3%
	% Magnitude	18.6%	-	23.8%	11.0%
Summary	Number	1673	1067	730	332

^a^: Compounds that do not fit into any of the above categories.

**Table 6:** Selected chemical components of Gd cottonseed kernels. Data are reported on a dry weight basis with average (A) and standard deviation (SD, n = 3). ADF, acid detergent fiber. ADL, acid detergent lignin. Adapted from [14].

**Major Component (g kg^−1^)**						
Moisture	Gossypol	Oil	Protein	ADF	ADL	Starch
67.9 ± 0.5	3.75 ± 0.02	387 ± 18	397 ± 8	100 ± 18	52.3 ± 10.1	12.2 ± 1.0
**Macro Element and Ash (g kg^−1^)**						
P	Ca	K	Mg	Na	S	Ash
9.8 ± 0.8	2.0 ± 0.3	12.0 ± 0.7	5.4 ± 0.4	0.6 ± 0.0	4.5 ± 0.3	46.7 ± 0.8

## Data Availability

The data presented in this study are available upon request.

## Sample Availability

Samples of the compounds are not available from the authors.
